# Hydrogen Peroxide-Induced Inhibition of Vasomotor Activity: Evaluation of Single and Combined Treatments With Vitamin A and Insulin in Streptozotocin-Diabetic Rats

**DOI:** 10.1080/15604280214484

**Published:** 2002

**Authors:** Fulya Zobali, Tanju Besler, Nuray Ari, Çimen Karasu

**Affiliations:** 1 Department of Pharmacology Faculty of Pharmacy Ankara University Ankara Turkey; 2 Department of Nutrition and Dietetics Faculty of Medicine Hacettepe University Ankara Turkey; 3 Department of Pharmacology Faculty of Pharmacy Ankara University Tandogan, Ankara 06100 Turkey

## Abstract

A positive correlation has been established between increased oxidative stress and cardiovascular diseases in diabetes mellitus. We evaluated the effects of single or combined treatments with vitamin A (retinol acetate, 30 mg/kg/day, for 12-weeks) and insulin (8-10 IU/rat/day for the final 6-week) on vasomotor activity, oxidative stress and retinol metabolism in 12-week streptozotocin diabetic rats. The vasomotor activity was determined by measuring in *vitro* responsiveness of aorta rings to phenylephrine (PE) and acetylcholine (ACh) in the absence or in the presence of hydrogen peroxide (H_2_O_2_). Preincubation with H_2_O_2_ (10 μM) produced a significant decrease in PE (1 mM)-induced contraction in untreated-diabetic but not in control rats. Single treatment with insulin counteracted this effect of H_2_O_2_ and also reversed the increased contractile response of diabetic aorta to PE, while vitamin A was found to be ineffective. H_2_O_2_ (10 μM) also inhibited ACh (1 mM)-stimulated endothelium- dependent relaxation two fold more in diabetic than in control aorta. In the prevention of H_2_O_2_-induced inhibition of vascular relaxation to ACh, vitamin A alone was markedly effective while insulin alone was not. The combination of vitamin A plus insulin removed the inhibitory action of H_2_O_2_ in diabetic aorta. Diabetic animals displayed an increased level of aorta thiobarbituric acid reactive substance (TBARS) in association with decreased levels of plasma retinol and retinol-binding protein (RBP). Single treatment with insulin, in spite of allowing recovery of normal growth rate and improved glucose and retinol metabolism in diabetic rats, was unable to control TBARS production to the same extent as vitamin A alone. Our findings suggest that the maintenance of ACh-stimulated endothelium-dependent vasorelaxant tone in normal physiological levels depends largely on the prevention and/or inhibition of peroxidative stress, which is achieved by combined treatment with vitamin A plus insulin. The use of vitamin A together with insulin provides a better metabolic control and more benefits than use of insulin alone in the reduction of diabetes-induced vascular complications.


Int. Jnl. Experimental Diab. Res., 3:119-130, 2002
© 2002 Taylor and Francis
1560-4284/02 $12.00 + .00

### Hydrogen Peroxide-induced Inhibition of Vasomotor Activity: Evaluation of Single and Combined Treatments with Vitamin A and Insulin in Streptozotocin-diabetic Rats

FULYA ZOBALI[1], TANJU BESLER[2], NURAY ARI[1], ÇIMEN KARASU[1]*

(Antioxidants in Diabetes-Induced Complications) The ADIC Study Group
[1] Department of Pharmacology, Faculty of Pharmacy, Ankara University, Ankara, Turkey
[2] Department of Nutrition and Dietetics, Faculty of Medicine, Hacettepe University, Ankara, Turkey

*Received: June 14, 2001; In final form: November 20, 2001*

A positive correlation has been established between increased oxidative stress and cardiovascular diseases in diabetes mellitus. We evaluated the effects of single or combined treatments with vitamin A (retinol acetate, 30 mg/kg/day, for 12-weeks) and insulin (8-10 IU/rat/day for the final 6-week) on vasomotor activity, oxidative stress and retinol metabolism in 12-week streptozotocin diabetic rats. The vasomotor activity was determined by measuring *in vitro* responsiveness of aorta rings to phenylephrine (PE) and acetylcholine (ACh) in the absence or in the presence of hydrogen peroxide ($H_2O_2$). Preincubation with $H_2O_2$ (10 μM) produced a significant decrease in PE (1 mM)-induced contraction in untreated-diabetic but not in control rats. Single treatment with insulin counteracted this effect of $H_2O_2$ and also reversed the increased contractile response of diabetic aorta to PE, while vitamin A was found to be ineffective. $H_2O_2$ (10 μM) also inhibited ACh (1 mM)-stimulated endothelium-dependent relaxation two fold more in diabetic than in control aorta. In the prevention of $H_2O_2$-induced inhibition of vascular relaxation to ACh, vitamin A alone was markedly effective while insulin alone was not. The combination of vitamin A plus insulin removed the inhibitory action of $H_2O_2$ in diabetic aorta. Diabetic animals displayed an increased level of

*Corresponding author: Department of Pharmacology, Faculty of Pharmacy, Ankara University, 06100, Tandogan, Ankara, Turkey; Phone: (+90) 312 2126805; Fax: (+90) 312 2131081; e-mail: karasu@pharmacy.ankara.edu.tr

**aorta thiobarbituric acid reactive substance (TBARS) in association with decreased levels of plasma retinol and retinol-binding protein (RBP). Single treatment with insulin, in spite of allowing recovery of normal growth rate and improved glucose and retinol metabolism in diabetic rats, was unable to control TBARS production to the same extent as vitamin A alone. Our findings suggest that the maintenance of ACh-stimulated endothelium-dependent vasorelaxant tone in normal physiological levels depends largely on the prevention and/or inhibition of peroxidative stress, which is achieved by combined treatment with vitamin A plus insulin. The use of vitamin A together with insulin provides a better metabolic control and more benefits than use of insulin alone in the reduction of diabetes-induced vascular complications.**

*Key words:* STZ-diabetes, rat aorta, hydrogen peroxide, vasomotor activity, vitamin A, insulin, retinol binding protein, peroxidative stress

### INTRODUCTION

One of the most dangerous consequences of diabetes-associated "glucose toxicity" is oxidative stress resulting from vascular complications [1-3]. We consistently investigated the role of reactive oxygen species in abnormal vascular reactivity and endothelial function in experimental diabetes [4-7]. Our previous findings suggested that increased production of superoxide anion radical ($^{\bullet}O_2^{-}$) associates with increased vasoconstrictor response to α-adrenoceptor agonists and accompanies the enhanced transient endothelium-dependent vasorelaxation in diabetic animals [4,7,8]. In addition to $^{\bullet}O_2^{-}$, hydrogen peroxide ($H_2O_2$) is also an important candidate responsible for the development of diabetes-induced vascular disease since its increased vascular production and activity was reported in diabetic vessels [6,7]. Our investigations have also focused on exploring the benefits of various antioxidants in the prevention or recovery of oxidative stress-induced cardiovascular abnormalities in diabetes mellitus. We have reported that the treatment with vitamin E [4,5,9], probucol [10] and alpha-lipoic acid [11] serve as important therapeutic approach against diabetes-induced vascular complications.

Vitamin A (retinol), is a member of the lipid-soluble retinoid compounds, plays a central role in the maintenance of many essential biological processes, and shows a function as lipoperoxyl-radical scavenger [12,13]. It has been reported that long-term vitamin A deficiency and poorly controlled insulin-dependent diabetes mellitus (IDDM) share common complications such as blindness, impaired immune response and increased risk of ischemic heart disease [14]. Abnormal metabolism of vitamin A, as indicated by its decreased circulatory levels along with carrier protein, retinol-binding protein (RBP), has been reported to occur in IDDM [14,15] and STZ-induced diabetic rats [16] but not in non-insulin dependent diabetes mellitus (NIDDM) [17]. It has been also reported that in subnormal vitamin A status in poorly controlled diabetic subjects or diabetic animals there is no response to vitamin A supplementation [14]. I*n vivo* insulin treatment is able to reverse abnormal vitamin A metabolism [14] as well as impaired vascular reactivity associated with diabetes [8]. On the other hand, vitamin A supplementation has been reported to modulate the negative effects of dietary fat on aorta responses to acetylcholine (ACh) [18] and to preserve endothelial vasodilator function through a mechanism related to vascular tissue antioxidant content in atherosclerotic rabbits [19]. Although increased risk of cardiovascular disease at suboptimal plasma concentrations of vitamin A [20] and decreased plasma concentrations of vitamin A and RBP in IDDM have previously been identified, the effects of vitamin A supplementation or the comparable effects of vitamin A with insulin on abnormal vasomotor activity of STZ-diabetic rats have not been investigated previously. Our study was undertaken to compare the effects of single or combined treatments with insulin and vitamin A on STZ-diabetes-induced alterations in vasomotor activity by the assessment of responsiveness of aorta rings to phenylephrine (PE) and ACh in the absence or in the presence of exogenous $H_2O_2$. We also assessed the general characteristics, plasma retinol and RBP levels of all experimental animals used in this study.

### MATERIALS AND METHODS

### *Animals, induction of diabetes and the treatment protocol*

"The principles of laboratory animal care" (NIH publication No. 85-23, revised 1985) were followed. Male Wistar rats weighing 250-300 g were used in the study. Diabetes was induced by 55 mg/kg, i.p. injection of STZ and confirmed by tail vein blood glucose levels two days later. Rats with blood glucose levels of 300 mg/dl or above were considered as diabetic. Two days after injection of either STZ or vehicle, rats were divided into following groups: 1) Untreated-diabetic rats (n = 15); 2) Diabetic rats treated with retinol acetate (30 mg/kg/day, orally) for 12 weeks (n = 12); 3) Diabetic rats untreated for 6 weeks and then treated with insulin (8-10 IU/rat/day, s.c.) for 6 weeks (n = 9); 4) Diabetic rats treated with both retinol acetate and insulin as same as protocols 2 and 3 (n = 9); 5) Untreated control rats (n = 15). The treatment dose of retinol acetate was chosen according to a previous study, and retinol acetate (2 mg) has been prepared into 0.1 ml corn oil as described previously [21]. Rats were maintained under standard housing conditions for 12 weeks before experiments were conducted.

### *Isolation of aortic rings and assessment of vasomotor activity*

Details of the method have been described previously [4-7]. Before the aorta was removed, samples of blood were obtained by cardiac puncture. Thoracic aorta was carefully cleaned from fat and connective tissue and was sectioned into 3-4 mm-long rings. In all rings, extreme care was taken to avoid stretching and contact with the luminal surface of endothelium during isolation. The rings were suspended between parallel hooks in 5 ml tissue baths filled with Krebs-Henseleit bicarbonate buffer solution, which were thermoregulated at 37 °C. The buffer consisted of (mM): NaCl 118, KCl, 4.7, $CaCl_2$ 2.5, $MgSO_4$ 1.2, $KH_2PO_2$ 1.2, $NaHCO_3$ 25 and glucose 11. Changes in isometric tension were recorded on an Ugo Basile recorder via Ugo Basile-7004 transducer (Varese, Italy). All rings were equilibrated for 60 min under a resting tension of 2 g before experiments were begun. During this period, the rings were washed every 15 min.

At the end of the equilibration period, concentration-response curves to increasing concentrations of PE were performed on each ring. Rings were then contracted with a submaximal concentration of phenylephrine, which produced approximately 80% of the maximum response. This concentration was usually 1 μM, but was occasionally varied between 1–3 μM to obtain equieffective agonist activity. After reaching plateau of contraction, cumulative concentration response curves to ACh (0.01–10 μM) were obtained to evaluate endothelium-dependent relaxation. In some rings, the vascular effect of a single dose of PE (1 μM) or ACh (1 μM) was evaluated in the absence or in the presence of $H_2O_2$ (1 μM - 1 mM, 30 min preincubation).

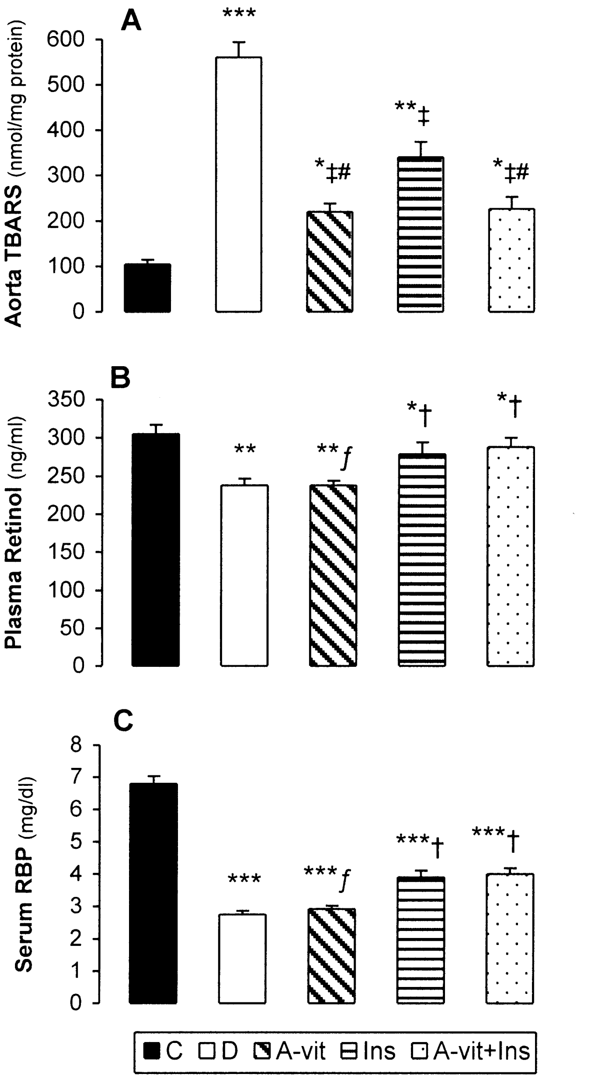

FIGURE 1
Thiobarbituric acid reactive substance in aorta homogenates (**A**), plasma retinol (**B**) and serum retinol binding protein (RBP) (**C**) levels in untreated-control (C), untreated-diabetic (D), vitamin A treated-diabetic (A-vit), insulin treated-diabetic (Ins.) and vitamin A plus insulin treated-diabetic (A-vit+Ins.) rats. Data (mean ± SEM) were analysed by ANOVA and between groups differences for each variable were tested using Newman-Keul's test. **P*<0.05, ***P*<0.01, ****P*<0.001 *vs*. control; †*P*<0.05, ‡*P*<0.001, *vs*. untreated-diabetic; *f*P<0.05, #*P*<0.01 *vs*. insulin-treated diabetic rats.

### *Food and water consumptions, body weights, blood and tissue analysis*

Food and water consumptions were measured daily over a 24-h period for each cage of three rats, but data were transformed to give values/rat/day. Body weights of animals were recorded at least once a week. Blood glucose concentrations were measured weekly by an Ames glucometer (Glucometer III, Bayer Diagnostics, France).

Plasma retinol concentrations were determined by a high-performance liquid chromatography as previously described [22]. For measuring serum retinol binding protein (RBP), a "radial immunodiffusion assay" method was used [23].

Thiobarbituric acid reactive substances (TBARS), as an index of lipid peroxidation hence oxidative stress were measured by a previously described method [24] in aorta homogenates.

### *Drugs and Statistical analysis*

All chemicals were purchased from Sigma Chemical (St. Louis, MO, USA). Food and water intake, although expressed per rat, were calculated per cage, so that statistical analysis was not legitimate. Contractile response to phenylephrine was expressed as gram tension/mg tissue wet weight. Relaxations to cumulative doses of ACh were expressed as a percentage decrease of the maximum contractile response. The sensitivity to the agonist was evaluated as the $pD_2$ (– log $EC_{50}$). Data are expressed as mean ±SE. They were first subjected to Bartlett's test for homogeneity of variances and were given a log transformation if necessary. One-way analysis of variance was then performed, followed by the Student-Newman-Keuls test to estimate the significance of differences for individual between-group comparisons. For the interaction study, differences within group before and after $H_2O_2$ incubation were analysed using unpaired Student's *t* tests. A value of *P* less than 0.05 was considered statistically significant.

TABLE 1
Table shows daily food and water consumptions, body weights and blood glucose levels of untreated-control (**Control**), untreated-diabetic (**Diabetic**), vitamin A treated-diabetic (**Diabetic + vitamin A**), insulin treated-diabetic (**Diabetic + insulin**) and vitamin A plus insulin treated-diabetic (**Diabetic + vitamin A and insulin**) animals. The maximum contractions to phenylephrine, maximum relaxation to acetylcholine and $pD_2$ values for phenylephrine or acetylcholine are also demonstrated in this table. $pD_2$ values calculated from phenylephrine or acetylcholine concentration-response curves. Data (mean ± SEM) were analysed by ANOVA and between groups differences for each variable were tested using Newman-Keul's test. Mean daily food and water consumptions although expressed per rat, were calculated per cage, so that statistical analysis was not legitimate. 1-week: 1-week after STZ injection. 6-week: 6-week after STZ injection. 12-week: 12-week after STZ injection; at the end of the treatment period. $^aP<0.05$, $^bP<0.01$, $^cP<0.001$ *vs.* control; $^dP<0.05$, $^eP<0.01$, $^fP<0.001$ *vs.* diabetic.

| | **Control** (n=15) | **Diabetic** (n=15) | **Diabetic + Vitamin A** (n=12) | **Diabetic + Insulin** (n=9) | **Diabetic + Vitamin A and Insulin** (n=9) |
|---|---|---|---|---|---|
| Daily food consumption (g) | | | | | |
| 1-week | 23.4 ± 0.54 | 31.2 ± 0.40 | 31.2 ± 0.45 | 32.4 ± 0.44 | 30.8 ± 0.34 |
| 6-week | 25.1 ± 0.69 | 40.1 ± 0.68 | 36.2 ± 0.71 | 39.1 ± 0.22 | 37.3 ± 0.31 |
| 12-week | 25.3 ± 0.50 | 47.6 ± 1.40 | 37.8 ± 0.43 | 31.1 ± 0.80 | 27.2 ± 0.90 |
| Daily water consumption (ml) | | | | | |
| 1-week | 25.4 ± 1.0 | 109 ± 20 | 112 ± 18 | 100 ± 14 | 105 ± 10 |
| 6-week | 30.0 ± 0.9 | 250 ± 13 | 210 ± 5.0 | 250 ± 6.0 | 229 ± 3.0 |
| 12-week | 42.3 ± 6.3 | 260 ± 14 | 152 ± 12 | 118 ± 11 | 85.0 ± 9.0 |
| Body weight (g) | | | | | |
| 1-week | 277 ± 17 | 248 ± 5.3 | 272 ± 15 | 250 ± 8.3 | 273 ± 6.7 |
| 6-week | 344 ± 15 | 219 ± 14 $^c$ | 220 ± 9.0 $^c$ | 217 ± 4.7 $^c$ | 218 ± 9.3 $^c$ |
| 12-week | 367 ± 10 | 152 ± 8.0 $^c$ | 188 ± 4.0 $^{c,d}$ | 230 ± 6.5 $^{c,e}$ | 266 ± 4.1 $^{c,f}$ |
| Blood glucose (mM) | | | | | |
| 1-week | 5.1 ± 0.1 | 17.9 ± 0.2 $^c$ | 16.4 ± 0.4 $^c$ | 18.3 ± 0.5 $^c$ | 17.0 ± 0.5 $^c$ |
| 6-week | 4.9 ± 0.1 | 26.3 ± 0.3 $^c$ | 21.9 ± 0.7 $^c$ | 25.2 ± 0.6 $^c$ | 21.4 ± 0.6 $^c$ |
| 12-week | 4.8 ± 0.1 | 26.6 ± 0.8 $^c$ | 20.0 ± 0.8 $^{c,d}$ | 12.4 ± 1.5 $^{b,f}$ | 8.1 ± 1.3 $^{a,f}$ |
| Maximum contraction to phenylephrine (g tension/mg tissue) | 0.143 ± 0.01 | 0.266 ± 0.01 $^c$ | 0.204 ± 0.008 $^{b,e}$ | 0.158 ± 0.01 $^f$ | 0.155 ± 0.01 $^f$ |
| $pD_2$ values for phenylephrine | 6.90 ± 0.16 | 7.05 ± 0.21 | 7.03 ± 0.28 | 6.89 ± 0.33 | 7.10 ± 0.25 |
| Maximum relaxation to acetylcholine (%) | 91.2 ± 0.99 | 87.6 ± 1.38 | 88.2 ± 2.70 | 92.1 ± 3.26 | 90.9 ± 1.24 |
| $pD_2$ values for acetylcholine | 7.33 ± 0.34 | 7.14 ± 0.25 | 7.31 ± 0.31 | 7.25 ± 0.24 | 6.99 ± 0.40 |

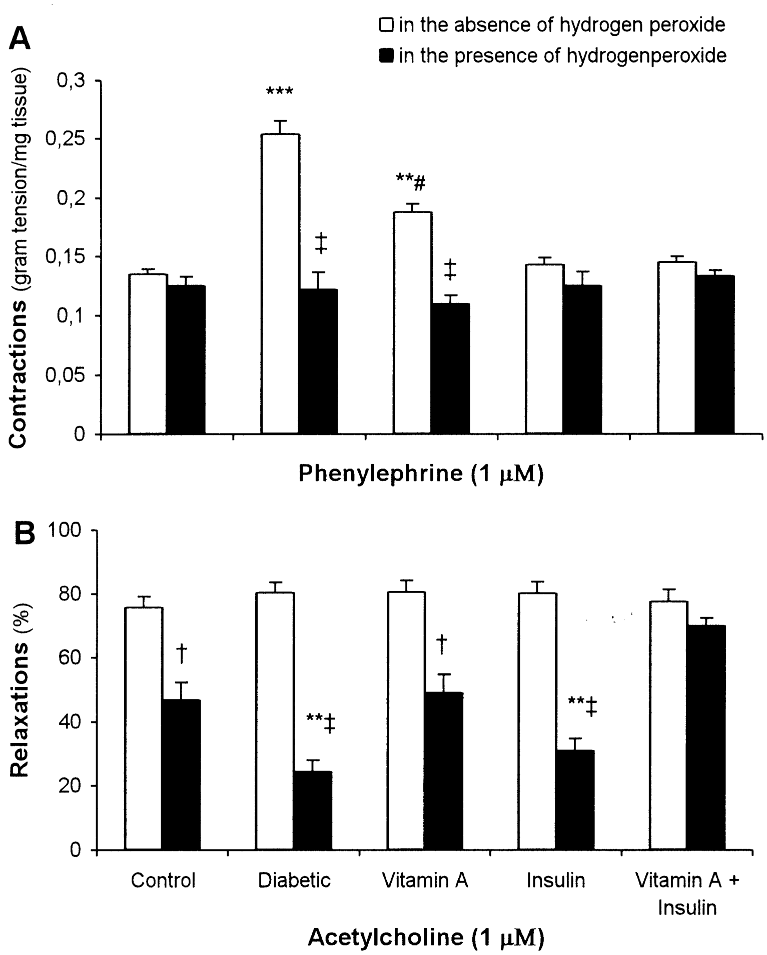

FIGURE 2
The contractile response of aorta to 1μM concentration of phenylephrine (**A**) and the relaxant response of aorta to 1μM concentration of acetylcholine (**B**) in the presence (dark) or in the absence (light) of $H_2O_2$ (10 μM) in untreated-control (control, n=10), untreated-diabetic (diabetic, n=10), vitamin A treated-diabetic (vitamin A, n=9), insulin treated-diabetic (insulin, n=9) and vitamin A plus insulin treated-diabetic (vitamin A + insulin, n=8) rats. Data (mean ± SEM) were analysed by ANOVA and between groups differences for each variable were tested using Newman-Keul's test. *$P<0.05$, **$P<0.01$, ***$P<0.001$ *vs.* control; $^{\#}P<0.01$ *vs.* untreated-diabetic. $^{\dagger}P<0.01$, $^{\ddagger}P<0.001$ express statistically significant difference before and after $H_2O_2$ within each group.

### RESULTS

### *Food and water consumption, body weights, and blood glucose levels*

As expected, the food and water intake was increased in untreated-diabetic animals (Table 1). The single treatment with insulin was more effective than vitamin A treatment in the recovery of food and water intake of diabetic animals. However, combined treatment with vitamin A plus insulin almost entirely controlled feeding regimen of diabetic animals (Table 1). The effects of both treatments were progressive. The body weight loss was more effectively controlled by single insulin treatment when compared with the effect of single vitamin A treatment. Although the combined treatment

The percentage inhibition in PE-induced contraction in the presence of $H_2O_2$ was 17.9 ± 4.57% in insulin-treated diabetic animals, and 15.5 ± 4.07% in insulin plus vitamin A treated-diabetic animals for both P>0.05 *vs.* non-diabetic control rats; P<0.001 *vs.* untreated-diabetic rats.

Preincubation of rings with $H_2O_2$, at 10 µM, inhibited 1 µM ACh-induced endothelium-dependent relaxation in non-diabetic control rats (38.6 ± 6.01%) (Figure 2B). This effect of $H_2O_2$ significantly increased in untreated-diabetic rats (74.5 ± 4.07%; P<0.001 *vs.* non-diabetic control rats). In rats treated with vitamin A alone, $H_2O_2$–induced inhibition in ACh response was prevented and the degree of $H_2O_2$–induced inhibition was observed very similar with the corresponding parameter from the non-diabetic control animals (34.1 ± 5.00%; P>0.05 *vs.* non-diabetic control group; P<0.01 *vs.* untreated-diabetic group). Single treatment with insulin did not change the inhibitory action of $H_2O_2$ (69.04 ± 4.61%; P<0.001 *vs.* non-diabetic control group; P>0.05 *vs.* untreated-diabetic group). The inhibitory action of $H_2O_2$ on endothelium-dependent relaxations disappeared when diabetic animals were treated with vitamin A plus insulin (18.41 ± 4.75%; P>0.05 *vs.* non-diabetic control and P<0.001 *vs.* untreated-diabetic animals) (Figure 2B).

A preliminary study of normal age-matched rats was carried out to determine the effects of treatments with vitamin A. Since no change in vascular responses or blood and tissue analysis was observed, data are not shown. Normal control animals were not treated with insulin because of hypoglycemia risk.

### DISCUSSION

Our confirmed previous investigations showing that the STZ-diabetes results in increased vascular reactivity to α-adrenoceptor agonists [2,4,8,25-27]. The diabetes-induced increased vasoconstrictor activity has been linked with excess production of reactive oxygen species [2-4,7]. I*n vivo* treatments with insulin [8,27,28] or other antioxidants [4,11,29] were demonstrated to prevent or reverse the abnormal contractile response in diabetic vessels. Previously, we found that *in vivo* treatment with a low dose of insulin (4 IU/kg/day, for 15 days) is able to reverse the increased vasoconstrictor response to norepinephrine in 6 weeks alloxan-diabetic rats [8]. Accordingly, present study demonstrated that the increased PE-induced tone is completely reversed by a higher dose of insulin (8-10 IU/rat/day, for 6 weeks, started after 6-week of untreated-diabetes) in 12 weeks STZ-diabetic rats. This insulin regimen also well controlled hyperglycemia and vitamin A metabolism, and partly decreased oxidative stress in diabetic animals. Other investigators showed that insulin treatment improved the increased protein kinase C activity and diacylglycerol levels [30] that are responsible for abnormal vascular reactivity to α-adrenoceptor agonists in diabetic vessels. In our study, single treatment with vitamin A throughout 12-week showed less effectiveness than insulin monotreatment in controlling vasoconstrictor activity and hyperglycemia in diabetic rats. In addition, antioxidant treatment with vitamin A, started two days after STZ injection was unable to maintain the normal metabolism of endogenous retinol; however it markedly reduced the oxidative stress in diabetic vessels. Our findings indicate that the replacement of insulin hence the management of hyperglycemia as well as other metabolic events are essential to achieve normal contractile response of vessels to the stimulants of α-adrenoceptors in an insulin-dependent model of diabetes. To our knowledge, the effects of treatment with vitamin A alone or combination of vitamin A with insulin on vasomotor activity in diabetic vessels have not been studied previously. Nevertheless, dietary vitamin A supplementation has been shown to reduce the maximal vessel tension in response to PE in rats fed different oil diets [18].

Another important finding of this study is that exogenous $H_2O_2$ at 10 mM inhibited PE-induced contraction in diabetic but not in control rats. When the incubation dose of $H_2O_2$ was increased to 1 mM, this inhibition was also observed in some non-diabetic control vessels. Although there is no previous report indicating depressor effect of $H_2O_2$ on PE-induced contraction in diabetic vessel, our findings are consistent with some previous studies showing that incubation with $H_2O_2$ at 1 mM, but not at 0.1 mM, is able to inhibit PE-induced contraction in non-diabetic normal rats [31,32]. These investigators explained the mechanism responsible for inhibitory action of $H_2O_2$ via impairment of the cellular signalling reactions initiated by the PE. In addition to these studies, recently, addition of 0.5 mM $H_2O_2$ to organ bath has been reported to cause a decrease in KCl-induced contraction in pig coronery artery [33]. The reason for increased destructive effect of $H_2O_2$ on PE-induced contraction in diabetic vessels could be related to its increased production and effectiveness in diabetic vessels. Previously, we reported that the biological availability and the vasodilator action of $H_2O_2$ are increased in diabetic aorta [6,7]. The increased responsiveness of diabetic vessels to the inhibitory actions of exogenous $H_2O_2$ may reflect an important facet of $H_2O_2$ linked to its specific modulatory role that may function to compensate increased vascular reactivity in response to $\alpha$-adrenoceptor stimulation under oxidative stress. It is well known that $H_2O_2$ produces vasorelaxation in PE-precontracted artery rings, and the relaxant effect of $H_2O_2$ requires formation of cyclic guanosine monophosphate (cGMP), and is mediated by nitric oxide (NO) [6,32,34,35]. In addition, $H_2O_2$ also augments the secretion of other endothelium-derived relaxing peptides, adrenomodulin and C-type natriuretic peptide, that has been reported to function to compensate the vasoconstriction in situations by which oxidative stress are increased, as in the case of hypertension and atherosclerosis [36].

Our study showed that the maximum endothelium-dependent relaxations of aortic rings to ACh are unaffected from of STZ-diabetes, insulin or vitamin A therapy. This is in accord with previous studies showing that 8-12 weeks STZ diabetes [7,34] or an antioxidant, $\alpha$-lipoic acid, [11] did not change endothelium-dependent vascular relaxations of rat aorta. Parallel with findings of other investigators [32], the incubation with $H_2O_2$ in our study produced a decrease in ACh-stimulated endothelium-dependent relaxation in the rat aorta. Additionally, we also demonstrated that the inhibitory action of $H_2O_2$ on ACh-induced relaxation significantly increased in diabetic animals. This effect of $H_2O_2$ may appear when the vessels are exposed long-term to the high concentrations of $H_2O_2$ as in the case of diabetes [6,7]. Some studies demonstrated the inhibitory actions of high concentrations of $H_2O_2$ on soluble guanylate cyclase [37] and endothelial nitric oxide synthase (eNOS) [38]. Also, a long-term treatment with $H_2O_2$ by luminal perfusion or in the organ bath was reported to result in loss of endothelium in arteries [32,33]. Long-term exposure of vascular smooth muscle cells [39] and endothelial cells [40] to $H_2O_2$ were reported to lead to sustained increase in intracellular calcium levels. It is well known that the final outcome of a sustained elevation of intracellular calcium causes tissue injury via lipid peroxidation and activation of calcium-dependent proteases [39].

In our study, insulin treatment of diabetic animals did not change the inhibitory action of $H_2O_2$ on ACh-induced relaxation. In contrast, vitamin A treatment alone prevented the inhibitory action of $H_2O_2$ on ACh-induced relaxation in diabetic animals. This is in agreement with a previous report showing the protective effect of vitamin A against $H_2O_2$–induced tissue toxicity [41]. Surprisingly, the inhibitory action of $H_2O_2$ disappeared and the ACh-induced relaxation normalized when insulin and vitamin A were given together to the diabetic animals, suggesting that insulin increases effectiveness of vitamin A against $H_2O_2$-induced destruction on eNOS activity / NO bioavailability. The underlying mechanism for the interaction of vitamin A with insulin is not completely known with total certainty, nevertheless previous studies have demonstrated that Type-1 diabetic patients or diabetic rats exhibit an impaired metabolic availability of vitamin A, and insulin treatment reverses to normal the diabetes-induced changes in vitamin A metabolism [14,16,42]. Accordingly, our study showed that *in vivo* insulin treatment improves abnormal metabolism of vitamin A. Vitamin A treatment also produced partially low blood glucose in untreated-diabetic animals. This finding is parallel with the previous reports showing that 1) some retinyl esters and vitamin A protect beta cells against STZ- and alloxan induced toxicity [43,44], and 2) vitamin A deficiency leads to an inhibition in insulin secretion *in vivo* and *in vitro* [43].

In our study, there was no significant difference in vessel TBARS levels between vitamin A-treated and vitamin A plus insulin treated diabetic animals. The TBARS lowering effect of insulin alone was moderate when compared with the effect of vitamin A alone. Although vitamin A plus insulin treatment did not provide a complete inhibition in TBARS levels, our findings suggest that the maintenance of ACh-stimulated endothelium-dependent vasorelaxant tone in normal level depends largely on the prevention and/or inhibition of peroxidative stress, which is achieved only by combined treatment with vitamin A plus insulin.

Insulin treatment alone ameliorated the endogenous retinol metabolism, better controlled blood glucose and weight gain and normalized PE-induced contractility in diabetic animals However, vitamin A treatment alone largely inhibited diabetes-induced oxidative/ peroxidative stress and its effects on endothelium-dependent relaxation. Combination of two agents especially is needed to maintain of ACh-stimulated endothelium-dependent vasorelaxant tone in normal physiological level. In conclusion, when considering the achievements of the two agents in different steps of controlling diabetes-induced metabolic and vascular abnormalities, the combination of insulin with an antioxidant, vitamin A, appears to be a more appropriate strategy than insulin monotherapy in the treatment of diabetic complications.

### REFERENCES

1. Ruderman, N.B., Williamson, J.R. and Brownlee, M. (1992) Glucose and diabetic vascular disease, *FASEB J,* **6**, 2905-2914.
2. Chang K.C., Chung S.Y., Chong W.S., Suh J.S., Kim S.H., Noh H.K., Seong B.W., Ko H.J. and Chun K.W. (1993) Possible superoxide radical-induced alteration of vascular reactivity in aortas from streptozotocin-treated rats, *J Pharmacol Exp Therap,* **266**, 992-1000.
3. Giugliano D., Ceriello A. and Paolisso G. (1996) Oxidative stress and diabetic vascular complications, *Diabetes Care,* **19**, 257-267.
4. Karasu, Ç., Ozansoy, G., Bozkurt, O., Erdogan, D. and Ömeroglu, S. (1997) Antioxidant and triglyceride lowering effects of vitamin E associated with the prevention of abnormalities in the reactivity and morphology of aorta from streptozotocin-diabetic rats, *Metabolism,* **46**, 872-879.
5. Karasu, Ç., Ozansoy, G., Bozkurt, O., Erdogan, D. and Ömeroglu, S. (1997) Changes in isoprenaline-induced endothelium-dependent and –independent relaxations of aorta in long-term STZ-diabetic rats: Reversal effect of dietary vitamin E, *Gen Pharmac,* **29**, 561-567.
6. Karasu Ç. (1999) Increased activity of $H_2O_2$ in aorta isolated from chronically streptozotocin-diabetic rats: effects of antioxidant enzymes and enzymes inhibitors, *Free Radic Biol Med,* **27**, 16-27.
7. Karasu Ç. (2000) Time course of changes in endothelium-dependent and -independent relaxation of chronically diahas more beneficial, the body weights of diabetic rats were still significantly lower than that in control rats at the end of treatment periods (Table 1).

The insulin or vitamin A treatment alone resulted in a significant fall in blood glucose levels of diabetic animals. However, the effect of vitamin A was fairly modest when compared with the effect of insulin alone. Combination of insulin with vitamin A was more effective in the controlling of hyperglycemia, but was unable to produce a normoglycemia in diabetic animals (Table 1).

### *THIOBARBITURIC ACID REACTIVE SUBSTANCES, RETINOL AND RETINOL BINDING PROTEIN LEVELS*

STZ-diabetes led to an increase in TBARS levels of aorta (Figure 1A). Single treatment with vitamin A significantly prevented TBARS augmentation; however a complete normalization was not achieved by this treatment. There was no significant difference in aorta TBARS levels between single vitamin A treatment and vitamin A plus insulin treatment. Single treatment with insulin was able to decrease TBARS levels in diabetic aorta, but its effect was moderate when compared with the effect of vitamin A alone or combined treatment with the two agents (Figure 1A).

Plasma retinol levels were significantly lower in untreated-diabetic animals than in non-diabetic control animals (Figure 1B). Insulin treatment either alone or in combination with vitamin A produced a significant increase in plasma retinol levels of diabetic animals. The effect of combination was very similar to the effect of insulin alone (Figure 1B). Plasma retinol levels was not changed when diabetic animals were treated with only vitamin A (Figure 1B).

Serum retinol binding protein (RBP) levels were profoundly decreased in untreated-diabetic rats (Figure 1C). Vitamin A treatment alone failed to affect serum RBP levels of diabetic rats. Diabetes-induced diminish in RBP was gently restored by insulin treatment. There was no evidence for an additive effect of two agents when applied together (Figure 3B).

### *VASOMOTOR ACTIVITY MEASUREMENTS*

The contractile response to cumulatively increasing concentrations and a single concentration (1 μM) of phenylephrine (PE) was significantly increased in untreated-diabetic rats (Table 1). Neither STZ-diabetes nor each treatment protocol altered the sensitivity ($pD_2$) to PE (Table 1). Insulin treatment alone normalized the contractile response of diabetic aorta to PE (Table 1, Figure 1A). Vitamin A treatment showed a similar trend to insulin treatment decreasing vasoconstriction in diabetic animals, but its effect was moderate. There was no significant difference between single insulin treatment and combined treatment in the recovery of PE-induced response in diabetic aorta (Table 1, Figure 1A).

The endothelium-dependent vasodilator effect of acetylcholine (ACh) did not seriously alter in aortic rings from diabetic animals (Table 1, Figure 1B). The maximum relaxations to ACh were similar in all experimental groups. No change in $pD_2$ values for ACh among the experimental groups was detected in the present study (Table 1).

Addition of $H_2O_2$ to the bath medium, at 10 μM, inhibited PE (1 μM)-induced contraction in diabetic rats (Figure 2A). When aortic rings incubated with 1 mM $H_2O_2$, slight inhibition was also observed in some control animals (data not shown). With respect to percentage changes in 1 μM PE-induced response, $H_2O_2$ produced inhibition was 12.8 ± 4.57% in control animals. This value was not significantly different from the percentage changes in the absence of $H_2O_2$, and was increased to 51.5 ± 6.42% by 12 weeks of STZ-diabetes ($P<0.001$). Vitamin A monotherapy did not significantly alter the inhibitory action of $H_2O_2$ (46.6 ± 7.04%; $P>0.05$ *vs.* untreated-diabetic group).

betic aorta: role of reactive oxygen species, *Eur J Pharmacol,* **392,** 163-73.

8. Karasu Ç. and Altan V.M. (1993) The role of endothelial cells on the alterations in vascular reactivity induced by insulin-dependent diabetes mellitus: Effects of insulin treatment, *Gen Pharmacol,* **24,** 743-757.
9. Karasu Ç, Dewhurst M, Stevens EJ, and Tomlinson DR. (1995) Effects of anti-oxidant treatment on sciatic nerve dysfunction in streptozotocin-diabetic rats; comparison with essential fatty acids, *Diabetologia,* **38,**129-134.
10. Karasu Ç. (1998) Acute Probucol treatment partially restores vasomotor activity and abnormal lipid metabolism whereas morphological changes are not affected in aorta from long-term STZ-diabetic rats, *Exp Clin Endocrinol Diabetes,* **106,** 189-196.
11. Koçak G, Aktan F, Canbolat O, Özo?ul C, Elbe? ?, Yıldızoglu-Arı N and Karasu Ç. (2000) Alpha-lipoic acid treatment ameliorates metabolic parameters, blood pressure, vascular reactivity and morphology of vessels already damaged by streptozotocin-diabetes (The ADIC Study Group), *Diab Nutr Metab,***13,** 308-319.
12. Blomhoff, R. (1994) Transport and metabolism of vitamin A. *Nutr Reviews,* **52,** S13-S23.
13. Livrea, M.A., Tesoriere, L., Bongiorno, A., Pintaudi, A.M., Ciaccio, M. and Riccio, A. (1995) Contribution of vitamin A to the oxidation resistance of human low density lipoproteins, *Free Radic Biol Med,* **18,** 401-409.
14. Basu, T.K. and Basualdo, C. (1997) Vitamin A homeostasis and diabetes mellitus, *Nutrition,* **13,** 804-806.
15. Martinoli, L., Di Felice, M., Seghieri, G., Ciuti, M., De Giorgio, L.A., Gori, R., Anichini, R. and Franconi, F. (1993) Plasma retinol and alpha-tocopherol concentrations in insulin-dependent diabetes mellitus: their relationship to microvascular complications, *Int J Vitam Nutr Res,* **63,** 87-92.
16. Tuitoek, P.J., Ziari, S., Tsin, A.T., Rajotte, R.V., Suh, M. and Basu, T.K. (1996) Streptozotocin-induced diabetes in rats is associated with impaired metabolic availability of vitamin A (retinol), *Br J Nutr,* **75,** 615-622.
17. Sasaki, H., Iwasaki, T., Kato, S. and Tada, N. (1995) High retinol/retinol-binding protein ratio in noninsulin-dependent diabetes mellitus, *Am J Med Sci,* **310,** 177-181.
18. Lutz, M., Cortez, J. and Vinet, R. (1995) Effects of dietary fats, alpha-tocopherol and beta-carotene supplementation on aortic ring segment responses in the rat, *Int J Nutr Res,* **65,** 225-230.
19. Keany, J.F., Gazzino, J.M., Xu, A., Frei, B., Curran-Celentano, J., Shwaery, G.T., Loscalzo, J. and Vita, J.A. (1993) Dietary antioxidants preserve endothelium-dependent vessel relaxation in cholesterol-fed rabbits *Proc Natl Acad Sci USA,* **90,** 11880-11884.
20. Gey, K.F., Moser, U.K., Jordan, P., Stahelin, H.B., Eichholzer, M. and Ludin, E. (1993) Increased risk of cardiovascular disease at suboptimal plasma concentration of essencial antioxidants: an epidemiological update with special attention to carotene and vitamin C, *Am J Clin Nutr,* **57(suppl),** 787S-797S.
21. Beatriz, T. and Bhagwan, D.G. (1976) Microzommal enzyme inducers and hypervitaminossis A in rats, *Arch, Pathol, Lab, Med,* **100,** 100-105.
22. Nierenberg, D.W. and Lester, D.C. (1985) Determination of vitamins A and E in serum and plasma using a simplified clarification method and high-performance liquid chromatography. *J Chromatogr,* **345,** 275-284.
23. Malvy, D.J., Poveda, J.D., Debruyne, M., Burtschy, B., Dostalova, L. and Amedee-Manesme, O. (1993) Immunonephelometry and radial immunodiffusion compared for measuring serum retinol-binding protein, *Eur J Clin Chem Clin Biochem,* **31,** 47-48.
24. Jain, S.K. and Levine, S.N. (1995) Elevated lipid peroxidation and vitamin E-quinone levels in heart ventricles of streptozotocin treated diabetic rats, *Free Radic Biol Med,* **18,** 337-341.
25. Koya, D. and King, G.L. (1998) Protein kinase C activation and the development of diabetic complications, *Diabetes,* **47,** 859-866.
26. Yuichiro, J.S. and Ford, G.D. (1992) Superoxide stimulates IP3-induced $Ca^{2+}$ release from vascular smooth muscle sarcoplasmic reticulum, *Am J Physiol,* **262,** H114-H116.
27. MacLeod, K.M. (1985) The effect of insulin treatment on changes in vascular reactivity in chronic, experimental diabetes, *Diabetes,* **34,** 1160-1167.
28. Pogatsa, G., Koltai, M.Z., Hadhazy, P., Koszeghy, A. and Ballagi-Pordany, G. (1988) Insulin induced reversibility of altered responsiveness in femoral arterial bed of diabetic dogs, *Diabetes Res,* **9,** 41-45.
29. Keegan, A., Cotter, M.A. and Cameron, N.E. (1999) Effects of diabetes and treatment with the antioxidant alpha-lipoic acid on endothelial and neurogenic responses of corpus cavernosum in rats, *Diabetologia,* **42,** 343-350.
30. Inoguchi, T., Xia, P., Kunisaki, M., Higashi, S., Feener, E.P. and King, G.L. (1994) Insulin's effect on protein kinase C and diacylglycerol induced by diabetes and glucose in vascular tissues, *Am J Physiol,* **267,** E369-E379.
31. Iesaki, T., Okada, T., Shimada, I., Yamaguchi, H. and Ochi, R. (1996) Decrease in $Ca^{2+}$ sensitivity as a mechanism of hydrogen peroxide-induced relaxation of rabbit aorta, *Cardiovasc Res,* 31, 820-825.
32. Mian, K.B. and Martin, W. (1997) Hydrogen peroxide-induced impairment of reactivity in rat isolated aorta: potentiation by 3-amino-1,2,4-triazole, *Br J Pharmacol,* **121,** 813-819.
33. Walia, M., Sormaz, L., Samson, S.E., Lee, R.M. and Grover, A.K. (2000) Effects of hydrogen peroxide on pig coronery artery endothelium, *Eur J Pharmacol,* **21,** 249-253.
34. Zembowicz, A., Hatchett, R.J., Jacubowski, A.M. and Gryglewski, R.J. (1993) Involvement of nitric oxide in the endothelium-dependent relaxation induced by hydrogen peroxide in the rabbit aorta, *Br J Pharmacol,* **110,** 151-158.
35. Yang, Z., Zhang, A., Altura, B.T. and Altura, B.M. (1999) Hydrogen peroxide-induced endothelium-dependent relaxation of rat aorta involvement of $Ca^{2+}$ and other cellular metabolites, *Gen Pharmacol,* **33,** 325-336.

36. Chun, T.H., Itoh, H., Saito, T., Yamahara, K., Doi, K., Mori, Y., Ogawa, Y., Yamashita, J., Tanaka, T., Inoue, M., Masatsugu, K., Sawada, N., Fukunaga, Y. and Nakao, K. (2000) Oxidative stress augments secretion of endothelium-derived relaxing peptides, C-type natriuretic peptide and adrenomedulin, *J Hypertension,* **18,** 575-580.

37. White, A.A., Crawford, K.M., Patt, C.S. and Lad, P.J. (1976) Activation of soluble guanylate cyclase from rat lung by incubation or by hydrogen peroxide, *J Biol Chem,* **251,** 7304-7312.

38. Matoba, T., Shimokawa, H., Nakashima, M., Hirakawa, Y., Mukai, Y., Hirano, K., Kanaide, H. and Takeshita, A. (2000) Hydrogen peroxide is an endothelium-derived hyperpolarizing factor in mice, *J Clin Invest,* **106,** 1521-1530.

39. Geeraerts, M.D., Ronveaux-Dupal, M.F., Lemasters, J.L. and Herman, B. (1991) Cytosolic free $Ca^{2+}$ and proteolysis in lethal oxidative injury in endothelial cells, *Am J Physiol,* **261,** C889-C896.

40. Krippeit-Drews, P., Haberland, C., Fingerle, J., Drews, G. and Lang, F. (1995) Effects of $H_2O_2$ on membrane potential and $[Ca^{2+}]_i$ of cultured rat arterial smooth muscle cells, *Biochem Biophys Res Commun,* **209,** 139-145.

41. Llesuy, S., Milei, J., Picone, V., Gonzales Flecha, B., Beigelman, R. and Boveris, A. (1995) Effect of vitamins A and E on ischemia-reperfusion damage in rabbit heart, *Moll Cell Biochem,* **145,** 45-51.

42. Lu, J., Dixon, W.T., Tsin, A.T. and Basu, T.K. (2000) The metabolic availability of vitamin A is decreased at the onset of diabetes in BB rats, *J Nutr,* **130,** 1958-1962.

43. Chertow, B.S., Webb, M.D., Leidy, J.W. and Cordle, M.B. (1989) Protective effects of retinyl palmitate on streptozotocin- and alloxan-induced beta cell toxicity and diabetes in the rat, *Res Com Chem Pathol Pharmacol,* **63,** 27-44.

44. Kudriashov, B.A., Ul'ianov, A.M. and Tarasov, A. (1993) Prophylactic effect of vitamin A, neutralizing the development of experimental insulin-dependent diabetes in animals, *Vopr Med Khim,* **39,** 20-22.

